# Linking Neuroinflammation and Neurodegeneration in Parkinson's Disease

**DOI:** 10.1155/2018/4784268

**Published:** 2018-04-16

**Authors:** Géraldine Gelders, Veerle Baekelandt, Anke Van der Perren

**Affiliations:** Laboratory for Neurobiology and Gene Therapy, Department of Neurosciences, KU Leuven, Leuven, Belgium

## Abstract

Neurodegenerative diseases such as Parkinson's disease (PD) and Alzheimer's disease (AD) impose a pressing burden on our developed and consequently aging society. Misfolded protein aggregates are a critical aspect of several neurodegenerative diseases. Nevertheless, several questions remain unanswered regarding the role of misfolded protein aggregates and the cause of neuronal cell death. Recently, it has been postulated that neuroinflammatory processes might play a crucial role in the pathogenesis of PD. Numerous postmortem, brain imaging, epidemiological, and animal studies have documented the involvement of the innate and adaptive immunity in neurodegeneration. Whether these inflammatory processes are directly involved in the etiology of PD or represent secondary consequences of nigrostriatal pathway injury is the subject of intensive research. Immune alterations in response to extracellular *α*-synuclein may play a critical role in modulating Parkinson's disease progression. In this review, we address the current concept of neuroinflammation and its involvement in PD-associated neurodegeneration.

## 1. Introduction

To the present day, neurodegenerative diseases such as Parkinson's disease (PD) and Alzheimer's disease (AD) impose a pressing burden on our developed and consequently aging society. Increasing life expectancy implicates that a large part of the population becomes susceptible to neurodegenerative disorders [1]. The currently available treatment options for PD are limited, mainly symptomatic and associated with decreased efficacy and unwanted side effects over time. Therapeutic strategies acting on the underlying pathogenesis of the disease in order to slow down or block the disease progression, as well as reliable and sensitive tests for early diagnosis, represent a large unmet medical need. PD has long been considered as a sporadic disorder with an age-related increase in incidence without a clear etiology. Over the last 20 years however, new genetic insights from monogenic Mendelian forms of PD and more recently from genome-wide association studies (GWAS) have strengthened the evidence that PD has a considerable genetic component [2]. In addition, environmental factors have been proposed as important risk factors or triggers for PD [3].

**Figure 1:**
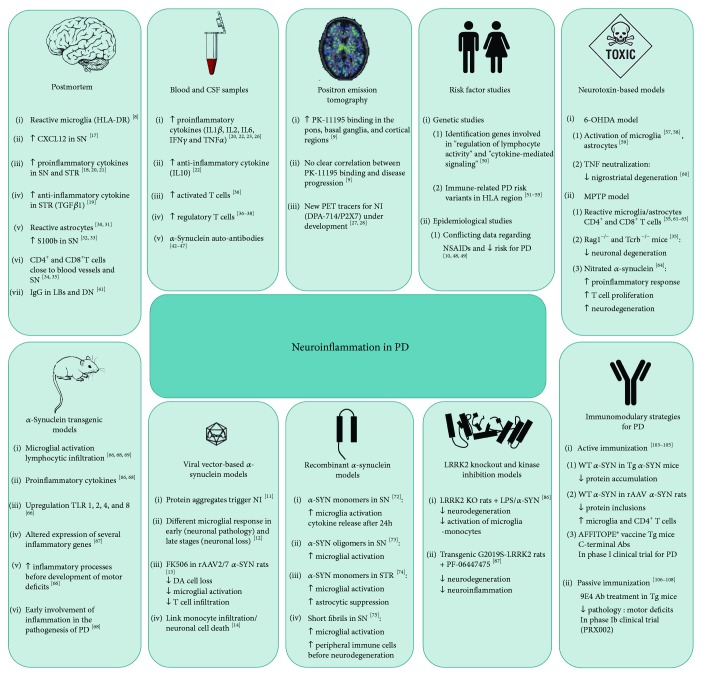
Overview of clinical and preclinical evidence linking neuroinflammation to neurodegeneration in Parkinson's disease. DN: dopaminergic neurons; TLR: toll-like receptor; STR: striatum; Tg: transgenic; PD: Parkinson's disease; KO: knockout; LBs: Lewy bodies; *α*-SYN: *α*-synuclein; NI: neuroinflammation.

The neuropathological hallmarks of PD are the deposition of misfolded protein aggregates, predominantly composed of *α*-synuclein, in distinct brain regions and the progressive degeneration of dopaminergic neurons in the substantia nigra pars compacta (SNpc), subsequently leading to striatal dopamine depletion, which is responsible for the classical motor symptoms [4, 5]. Throughout the last decade, this classical view has been extended to other nonmotor-related brain regions. It has become clear that the disease progresses following a characteristic pattern of pathological changes throughout the brain leading to severe nonmotor symptoms such as olfactory dysfunction, sleep disturbance, cognitive impairment, and autonomic dysfunction [6]. Nevertheless, several questions remain unanswered regarding the cause of neuronal cell death and the role of misfolded protein aggregates in PD. Multiple cellular and molecular mechanisms that might contribute to neuronal cell death have been described including mitochondrial dysfunction, oxidative stress, excitotoxicity, and proteasomal dysfunction. In addition, it has been postulated that neuroinflammatory processes might play a crucial role in the pathogenesis of PD and other neurodegenerative disorders [7]. Numerous postmortem, brain imaging, epidemiological, and animal studies have documented the involvement of the innate and adaptive immunity in neurodegeneration (schematic overview in Figure 1) [8–14]. Whether these inflammatory processes are directly involved in the etiology of PD or represent secondary consequences of nigrostriatal pathway injury is the subject of intensive research.

In this review, we address the current concept of neuroinflammation and its involvement in PD-associated neurodegeneration. We summarize our understanding of the involvement of innate and adaptive immunity in PD in patients as well as preclinical animal models. Lastly, we emphasize the current and potentially new therapeutic strategies targeting neuroinflammatory processes.

## 2. Evidence for Neuroinflammation in PD Patients

Typically, inflammation is a complex defense mechanism occurring in the body in response to perturbed homeostasis. The term “neuroinflammation” broadly defines the inflammatory processes occurring in the central nervous system (CNS) involving both the innate and adaptive immune system. This is a double-edged sword since the cells involved in neuroinflammatory responses can induce beneficial or harmful effects. Indeed, neuroinflammatory mechanisms contribute to both normal brain development and neuropathological events. Neuroinflammation has been repeatedly linked to neurodegeneration, including PD, over the last years, but whether the neuroinflammatory processes are a cause or a consequence of neuronal degeneration remains unanswered [7]. Two potential pathological mechanisms responsible for neuronal cell death exist in PD and most neurodegenerative disorders: “cell-autonomous” and “non-cell-autonomous” mechanisms. The cell-autonomous mechanism refers to an accumulation of intrinsic damage in the degenerating neurons resulting in their death. The second mechanism indicates an indirect degeneration of the affected neurons caused by pathological interactions with neighboring cells, such as resident glial cells (i.e., microglia and astrocytes) and/or infiltrating immune cells from the periphery (i.e., macrophages and lymphocytes).

Microglia are the principal innate immune cells in the brain and have fundamental roles in CNS homeostasis. They continuously scan their microenvironment and monitor ongoing synaptic activity (including synaptic pruning), clear apoptotic cells, and provide trophic support for neurons. They represent the first line of defense in the brain and react to pathological events through a cascade of inflammatory processes. In the presence of pathogens (pathogen-associated molecular patterns or PAMPS) or tissue damage (danger-associated molecular patterns or DAMPS), microglia induce complex immune responses by increasing the expression of toll-like receptors (TLRs) and several proinflammatory mediators, which consequently activate peripheral immune cells, with the aim of restoring tissue homeostasis [15, 16].

Earliest observations supporting the idea that neuroinflammatory processes are involved in PD date from postmortem studies 25 years ago. McGeer et al. reported the presence of human leukocyte antigen D-related- (HLA-DR-) positive reactive microglia in the SNpc of patients with PD [8]. Cytokines, chemokines, and other inflammatory mediators are known to trigger microglial activation, potentially contributing to nigrostriatal pathway injury. As dopaminergic neurons express a wide range of cytokine and chemokine receptors, it has been suggested that they are responsive to these inflammatory mediators which are derived from or which activate microglia. Accordingly, higher expression levels of the chemokine CXCL12 and its receptor CXCR4 were detected in the SN [17]. Elevated levels of the proinflammatory interleukins IL1*β*, IL2, IL6, and tumor necrosis factor *α* (TNF*α*) as well as the anti-inflammatory transforming growth factor *β*1 (TGF*β*1) have also been detected in the striatum, and concentrations of TNF*α*, IL1*β*, interferon *γ* (IFN*γ*), nitric oxide synthase (NOS), and reactive oxygen species (ROS) were found to be increased in the SN of postmortem samples [18–21]. These findings further confirm the involvement of microglia in the initiation of both pro- and anti-inflammatory events pointing towards the existence of multiple phenotypes in PD and distinct functions during disease progression. Aside from postmortem data, numerous *in vivo* studies have been conducted on biological fluids including blood and cerebrospinal fluid (CSF) of PD patients. Plasma and serum analysis showed upregulation of proinflammatory cytokines such as IL1*β*, IL2, IL6, IFN*γ*, and TNF*α* as well as the anti-inflammatory cytokine IL10 [22, 23]. Increased IL6 plasma concentrations were linked to a higher risk to develop PD [24]. An elevation in the serum levels of macrophage migration inhibitory factor (MIF) was also observed [25]. In line with these findings, the same proinflammatory cytokines (i.e., IL1*β*, IL6, and TNF*α*) have been reported in CSF samples [20, 26]. CSF is a valuable source of information for the discovery of new neurodegenerative and neuroinflammatory biomarkers as it mirrors metabolic and pathological alterations in the CNS more accurately than other biological fluids. In addition, noninvasive positron emission tomography (PET) imaging studies using PK-11195, a ligand of the peripheral benzodiazepine receptor (PBR; also known as the mitochondrial 18 kDa translocator protein or TSPO), which is selectively expressed by activated microglia, further confirmed the occurrence of microglial activation in PD [9]. PD patients showed significantly increased levels of PK-11195 binding in the pons, basal ganglia, and cortical regions. However, Gerhard et al. failed to find a clear correlation between PK-11195 binding and disease progression during a longitudinal follow-up study, suggesting that microglia might get activated early in the disease process [9]. Newer highly specific PET ligands (e.g., DPA-714 and P2X7) have been developed and are currently being tested to further confirm these imaging data [27, 28]. Which stimuli are responsible for this activation is currently under intensive debate, but abnormal misfolded proteins like extracellular *α*-synuclein have been proposed as one of the major candidates.

Besides microglia, astrocytes have also been found to participate in the neuropathology of PD [29, 30]. Astrocytes are resident cells from the brain contributing to the development and plasticity of the CNS, providing energy to neurons and maintaining brain homeostasis. In healthy individuals, astrocytes are heterogeneously distributed within the mesencephalon, with a low astrocytic density in the SN. In PD, an elevation in the number of astrocytes in the SN as well as pathological changes in astrocytes following a specific distribution pattern has been reported postmortem [30, 31]. Furthermore, upregulation of calcium-binding protein S100b, which is primarily expressed by astrocytes and acts as a cytokine, has been shown in the SN of postmortem PD patients. S100b may increase the expression of inducible nitric oxide synthase (iNOS) which, in turn, may result in the activation of the proinflammatory enzyme cyclo-oxygenase-2 (COX-2) in microglia as well as an increased production of nitric oxide (NO) and superoxide radicals [32, 33]. These events may directly or indirectly cause neuronal cell death. The role of astrocytes in the neuropathology of PD is still not well understood, since it has been hypothesized that these glial cells may prevent and/or exacerbate nigrostriatal injury due to a perturbed balance.

Over the last decades, the adaptive immune system has been shown to be involved in PD through the presence of CD4^+^ and CD8^+^ T lymphocytes in the vicinity of blood vessels and near dopaminergic neurons in postmortem brain tissues [34, 35]. On the other hand, B cells and natural killer cells were not detected [35]. Decreased serum levels of naïve lymphocytes were observed in PD patients while the numbers of activated T cells were increased, indicating that peripheral activation occurs in PD pathology [36]. Elevated numbers of regulatory T cells were also detected and exhibited an impaired ability to suppress the effector T cell function [36–38]. Recently, Sulzer and coworkers reported that well-defined *α*-synuclein peptides act as antigenic epitopes and drive CD4^+^ and CD8^+^ T cell responses in patients with PD [39]. The first antigenic region near the N-terminus, also referred to as the Y39 region, is strikingly close to the *α*-synuclein mutations causing PD (A30P, E46K, H50Q, G51D, and A53T) [40]. The second antigenic region encompasses S129 and requires S129 phosphorylation, a pathological form present in Lewy bodies. Further, they showed that T cells can respond to both *α*-synuclein epitopes arising from processing native *α*-synuclein present in the blood and fibrilized *α*-synuclein associated with PD. Orr et al. have described a role for humoral immunity in the pathogenesis of PD. They demonstrated a strong immunolabeling for IgG (immunoglobulin G) in Lewy bodies as well as one-third of the nigrostriatal dopaminergic neurons, implicating that IgG antibody coating labels these cells for degradation [41]. In the last years, the presence of auto-antibodies towards *α*-synuclein linking PD to humoral immunity has been investigated. These auto-antibodies are thought to be implicated in clearance pathways and tissue homeostasis, suggesting that they may play a crucial and protective role throughout disease progression [42]. Several studies based on the detection of *α*-synuclein auto-antibodies in biological fluids (i.e., blood, CSF, and saliva) using a wide range of assays, including electrochemistry, enzyme-linked immunosorbent assay (ELISA), immunoblotting, and surface plasmon resonance, have produced mixed results [42–47]. A plausible explanation could be interlaboratory variability regarding the assays and sample collecting procedures as well as the natural variation within patient cohorts. Therefore, additional studies overarching different patient groups with PD are indispensable.

Genetic and epidemiological studies have further implicated neuroinflammation as a risk factor in PD. Epidemiological studies based on the use of nonsteroidal anti-inflammatory drugs (NSAIDs) were conducted in order to investigate the involvement of neuroinflammation in the disease progression, with the underlying hypothesis that NSAIDs would decrease the risk of developing PD [10, 48, 49]. However, conflicting data were generated, possibly due to the fact that the NSAIDs were not clearly subdivided based on their working mechanism and pharmacokinetics. Therefore, more scrupulous studies regarding the possible correlation between the use of specific anti-inflammatory drugs and the risk of developing PD are required. More recently, pathway analysis-based GWAS have identified functional categories of genes involved in the “regulation of lymphocyte activity” and “cytokine-mediated signaling” providing support for a strong immune-related genetic susceptibility to PD [50]. The most obvious immune-related PD risk variants are located in the human leukocyte antigen (HLA) region (e.g., HLA-DRB1 and HLA-DRB5) [51–55]. HLA genes encode the major histocompatibility complex (MHC) proteins that are intimately involved in antigen presentation and immunity. This complex resides on the surface of antigen-presenting cells, including microglia, and promotes T cell activation. Altogether, these data point to the involvement of the innate immune system in PD. This is in line with findings from the AD field where GWAS have identified 20 well-validated genes harboring risk alleles (such as HLA-DRB1 and HLA-DRB5), of which about half are predominantly or only expressed in microglia [56].

## 3. Evidence from Neurotoxin-Based PD Models

Microglial activation has initially been observed in the SN and along the nigrostriatal tract in the classical 6-hydroxydopamine (6-OHDA) rat and mouse PD model [57, 58]. In addition, reactive astrocytes have been identified in the SN and the striatum of rats after exposure to 6-OHDA [59]. The 6-OHDA model is widely known to induce rather acute massive degeneration of dopaminergic neurons in the nigrostriatal pathway. McCoy et al. have shown that neutralization of the proinflammatory cytokine TNF led to reduced nigrostriatal degeneration in this model [60]. However, to investigate the involvement of peripheral immune and/or inflammatory components, proper controls are necessary as the blood-brain barrier (BBB) is transiently disrupted upon intracerebral injection of the neurotoxin. An alternative model that overcomes this issue is the systemic injection of 1-methyl-4-phenyl-1,2,3,6-tetrahydropyridine (MPTP). Reactive microglia and astrocytes have been detected in the MPTP-treated mouse and primate brain [61, 62]. Brochard et al. observed a selective nigrostriatal infiltration of CD4^+^ and CD8^+^ T cells in mice treated with MPTP, whereas no B lymphocytes were found [35]. It was postulated that the infiltration of T lymphocytes into the brain occurred after microglial activation but before astrogliosis. This model also provided evidence for the direct involvement of activated microglia in the processes leading to neuronal cell death as microglial activation precedes neuronal degeneration [35, 61, 63]. Furthermore, two immunodeficient mouse models lacking mature T lymphocytes (recombination activating gene 1^−/−^ (Rag1^−/−^) and T cell receptor beta chain^−/−^ (Tcrb^−/−^) mice) displayed a remarkably reduced susceptibility to MPTP-induced dopaminergic neurodegeneration [35]. Mice reconstituted with functional naïve lymphocytes partially lost their MPTP-related resistance, and CD4^+^ T cells were identified as the key players in this neurodegenerative process [35, 64]. Benner and coworkers hypothesized that protein oxidative modifications associated with PD (e.g., nitration of *α*-synuclein) might lead to novel antigenic epitopes able to initiate peripheral T cell responses that might consequently affect the nigrostriatal pathway [64]. Mice immunized with nitrated *α*-synuclein were shown to display marked proinflammatory responses and T cell proliferation. Adoptive transfer of T cells from mice immunized with nitrated *α*-synuclein to MPTP-treated mice led to a strong neuroinflammatory response as well as accelerated neuronal degeneration. These observations support the idea that CD4^+^ T cells are crucially implicated in neuroinflammation and subsequent neurodegeneration in animal models of PD. On the other hand, CD4^+^CD25^+^ regulatory T cells were shown to be neuroprotective through the suppression of microglia responses in the MPTP model [65].

## 4. Alpha-Synuclein: The Key Player in Neuroinflammation?

As the gene encoding for *α*-synuclein was the first to be unequivocally associated with a familial form of PD, *α*-synuclein transgenic mice have become one of the most studied genetic animal models for PD. However, since *α*-synuclein transgenic mice are in general hampered by a lack of dopaminergic cell loss and dopamine-dependent behavioral deficits, they may only provide partial evidence regarding the link between neuroinflammatory responses and genetic alterations. Watson and coworkers studied the temporospatial distribution of microglial activation and lymphocytic infiltration as well as the production of proinflammatory cytokines in mice overexpressing wild-type (WT) human *α*-synuclein under the neuron-specific Thy1-promoter [66]. Their study revealed an elevation in the number of IbaI^+^ reactive microglia and increased levels of TNF*α* in the striatum and later in the SN of young Thy1-*α*-synuclein mice. Microglial activation was maintained until 14 months of age, but increased serum levels of TNF*α* could only be detected until 5 to 6 months. Increased expression of TLR 1, 4, and 8, which potentially mediate microglial reactivity, was found in the SN of 5 to 6 months old animals, while TLR2 expression was elevated in the SN at 14 months. Serum levels of CD4^+^ and CD8^+^ T cells were upregulated at 22 months in the Thy1-*α*-synuclein mice reflecting the later role of the adaptive immunity in PD pathology. These results point to the occurrence of inflammatory processes before the development of motor deficits. In another study, three different *α*-synuclein transgenic mouse lines (WT and double A53T and A30P mutant human *α*-synuclein under the control of the tyrosine hydroxylase (TH) promoter and A53T human *α*-synuclein under the prion promoter) were used to investigate the molecular changes induced by *α*-synuclein [67]. The authors reported an altered expression of several genes, including inflammatory genes, in the SN of WT *α*-synuclein transgenic mice at stages preceding neuronal degeneration. In the mutant *α*-synuclein transgenic mice, in an advanced stage of the pathology, new candidate genes were identified that may be involved in protein deposition and neuronal cell death. Su et al. further documented the early involvement of both *α*-synuclein and inflammation in the pathogenesis of PD using mice overexpressing WT human *α*-synuclein driven by the rat TH promoter [68]. They observed an increase in the number of IbaI^+^ activated microglia in the SN as well as significantly elevated expression levels of TNF*α*. In line with these results, mice expressing the truncated form of human *α*-synuclein under the rat TH promoter also exhibited microglial activation in the SN [69].

Next to transgenic mouse models, recombinant adeno-associated viral vector- (rAAV-) based *α*-synuclein rodent and primate models, presenting various degrees of neuronal cell loss, have extended our view on neuroinflammatory events in PD pathogenesis. Theodore and coworkers noticed an increase in the numbers of CD68^+^ microglia and an expanding infiltration of B and T cells in the SN of rAAV2 *α*-synuclein mice before the onset of mild neuronal cell loss 6 months post injection [11]. The number of microglia declined 12 weeks post injection, but a persistent B and T cell infiltration was observed. Expression of proinflammatory cytokines was elevated, while the anti-inflammatory markers arginase I, IL4, and IL13 remained at the same level. These observations revealed that overexpression of *α*-synuclein, in the absence of overt neurodegeneration, is sufficient to initiate neuroinflammation. Sanchez-Guajardo et al. investigated in depth the early (neuronal pathology) and late stages (neuronal pathology in association with neurodegeneration) of the disease in a rAAV2/5 *α*-synuclein rat model [12]. A rapid and transient elevation in the number of microglia was shown after induction of neuronal pathology, resulting in a long-lasting activation of MHCII^+^ microglia. Neuronal pathology along with neuronal cell loss resulted in a delayed increase in the number of CD68^+^ microglia displaying a morphology similar to peripheral macrophages. Infiltration of T lymphocytes appeared to increase in function of the neurodegeneration-related severity. The authors concluded that the microglial response alters depending on the presence or absence of neuronal cell death implicating that microglia may exert different functions during the disease progression. Our group has shown that treatment with the immunophilin ligand FK506 improved the survival of dopaminergic neurons in a dose-dependent manner in a rAAV2/7 *α*-synuclein rat model [13]. Moreover, we demonstrated that FK506 led to decreased numbers of microglia/macrophages and lowered lymphocytic infiltration. These findings emphasize the anti-inflammatory properties of FK506 in reducing neurodegeneration as well as the causal role of neuroinflammatory processes in the pathogenesis of PD. In a recent publication, Harms et al. reported the necessary recruitment of peripheral CCR2^+^ monocytes to induce inflammation and subsequent neurodegeneration in a rAAV2 *α*-synuclein mouse model [14]. The role of the infiltrating monocytes in *α*-synuclein-mediated neuroinflammation and neurodegeneration was further investigated using the CCR2 knockout mouse model combined with *α*-synuclein overexpression. The authors found that the absence of infiltrating monocytes was neuroprotective compared to WT rAAV2 *α*-synuclein animals. These data suggest that the entry of peripheral monocytes into the brain might play a crucial role in *α*-synuclein-mediated neuronal cell death.

Evidence is emerging that *α*-synuclein can adopt distinct conformations or “strains” with remarkable differences in structural and phenotypic traits [70, 71]. Therefore, presentation of different *α*-synuclein assemblies (monomers, oligomers, or fibrils) to the innate immune system and the subsequent immune response might be a driving force in the disease, although different studies have not yet put forward one conformation as major determinant. Direct injection of recombinant monomeric *α*-synuclein in the mouse SN induced nigral microglial activation 24 hours post injection [72]. The proinflammatory cytokines IL1*β*, IL6, and TNF*α*, the proinflammatory enzyme COX-2, and the anti-inflammatory cytokine TGF*β* were upregulated in the SN compared to controls. Accordingly, strong microglial activation in the rat SN was found one week after injection of oligomeric (protofibrillar) *α*-synuclein [73]. Sznejder-Pachołek et al. have reported robust activation of microglia and elevated expression of IL1*α*, TNF*α*, and IFN*γ* in the striatum after striatal injection of monomeric *α*-synuclein. Also, an increase in striatal GFAP was shown four weeks post injection. These data further suggest that microglia and astrocytes are key players in *α*-synuclein-related neurotoxicity [74]. Very recently, Harms et al. have shown that short *α*-synuclein fibrils, but not monomeric *α*-synuclein, injected into the rat SN led to the formation of *α*-synuclein inclusions in dopaminergic neurons potentially spreading to the projection neurons in the striatum and rapidly induced microglial activation in the brain [75]. Axon loss was observed in the striatum along with the recruitment of monocytes two months post injection. Monocytes and macrophages initially displayed low MHCII expression in the striatum but, later, when *α*-synuclein inclusions were present in neighboring projection neurons, MHCII expression was strongly increased. These exciting results further support the hypothesis that recruitment of peripheral immune cells might happen before neurodegeneration.

**Figure 2:**
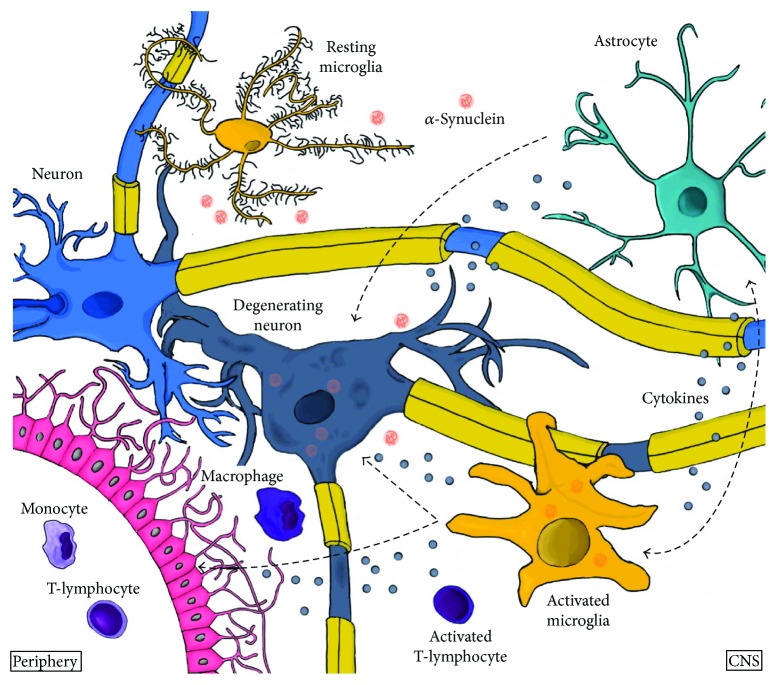
Possible link between *α*-synuclein, neuroinflammatory processes, and neurodegeneration in Parkinson's disease. In the presence of specific *α*-synuclein conformations, microglia get activated and induce a complex immune response by increasing the expression of toll-like receptors and several proinflammatory mediators, which consequently activates peripheral immune cells like monocytes or T cells. These peripheral immune cells might actively contribute to neurodegeneration.

Overall, these observations from animal models using distinct approaches implicate that the mechanisms leading to neuronal cell death involve nonneuronal cells (Figure 2). Molecular factors and mediators involved in these cellular interactions, including microglial activation as well as monocytic and lymphocytic infiltration, still need to be identified and characterized in order to find new targets for disease-modifying therapies.

## 5. LRRK2: *α*-Synuclein's Copilot in Neuroinflammation?

A second gene linked to autosomal dominant familial PD is the more recently discovered LRRK2 gene, encoding the leucine-rich repeat kinase 2 protein. Both LRRK2 and *α*-synuclein are key players in PD, but establishing a functional link between the two proteins has proven elusive. Interestingly, both proteins have been linked to microglial activation. Extracellular *α*-synuclein can directly initiate microglial activation and be phagocytosed by microglia, whereas LRRK2 has been reported to be involved in the intrinsic regulation of microglial activation as well as autophagolysosomal degradation [76, 77]. Genetic studies have also implicated LRRK2 in the development of autoimmune disorders including Crohn's disease and ulcerative colitis [78]. Microglia and monocytes display high LRRK2 expression levels, even higher than neurons, emphasizing its involvement in the innate immune system. This suggests that microglia and monocytes might be deeply involved in LRRK2-dependent processes and signaling (e.g., kinase activity and interactions with cofactors) [79, 80]. Indeed, several studies have shown that TLR2 and TLR4 stimulation results in the upregulation of LRRK2 expression and phosphorylation in primary microglia and monocytes [80, 81]. LRRK2 phosphorylation is currently thought to reflect the functional activity of the protein. Further, Russo et al. observed that LRRK2 deletion or pharmacological inhibition led to decreased production of proinflammatory mediators (i.e., IL1*β* and COX-2) and subsequent decline in the inflammatory response elicited by LPS or *α*-synuclein fibrils in microglial cells [82].

Over the last years, various studies using LRRK2 knockout or LRRK2 kinase inhibition in rodent models have been conducted to evaluate the effects of LRRK2 inactivation on neurodegeneration and neuroinflammation; however, conflicting data have been obtained. Lin et al. noticed a strong protection after genetic depletion of LRRK2 in a A53T *α*-synuclein CAMKII-promoter-driven transgenic mouse model [83]. In contrast, Herzig et al. found that high levels of both WT and G2019S-LRRK2 did not alter endogenous *α*-synuclein levels or exacerbate *α*-synucleinopathy in WT or A53T transgenic mice [84]. In line with these findings, Daher et al. reported that modulation of LRRK2 (i.e., deletion of LRRK2 or overexpression of human G2019S-LRRK2) in A53T *α*-synuclein transgenic mice had a minimal effect on *α*-synuclein-induced pathology in the mouse hindbrain, suggesting that these events are principally independent from LRRK2 expression [85]. Later, in line with the first study of Lin et al., it was shown that the LRRK2 knockout rat model was resistant to neuronal degeneration in association with a reduced activation of microglia and monocytes in response to LPS exposure or human WT *α*-synuclein overexpression [86]. While endogenous LRRK2 expression in the SN of WT rats is below detection level under physiological conditions, increased LRRK2 expression levels were found in the SN upon exposure to LPS or human WT *α*-synuclein overexpression. Based on these data, it has been suggested that knockdown of LRRK2 might be neuroprotective through the inhibition of chronically activated microglia as well as the recruitment of monocytes. These findings may provide valuable insights into the development of LRRK2-targeting therapeutic strategies. Indeed, LRRK2 kinase inhibitors are currently investigated with regard to their therapeutic potential as well as their efficacy and tolerability in *α*-synuclein models. PF-06447475, a LRRK2 kinase inhibitor, was administered for four weeks in both transgenic G2019S-LRRK2 and WT rats stereotactically injected with the rAAV2/1 vector expressing human WT *α*-synuclein [87]. G2019S-LRRK2 rats displayed aggravated degeneration of dopaminergic neurons and neuroinflammation upon *α*-synuclein overexpression, whereas treatment with PF-06447475 diminished neurodegeneration and neuroinflammation in these animals. Neuronal cell death was also found to be reduced in WT rAAV2/1 *α*-synuclein rats upon administration of PF-06447475. Further insights into the potential LRRK2-dependent regulation of microglial activation and neuroinflammation will provide valuable and crucial information.

## 6. Neuroinflammation beyond the CNS

The recently proposed prion-like behavior of *α*-synuclein may explain the observations made by Braak et al. implicating that the neuropathology of PD evolves in a patterned and sequential manner, with premotor symptoms preceding the presence of motor deficits [88, 89]. This pathological process is divided into six stages in PD initiating with *α*-synuclein-rich inclusions in the olfactory bulb and the dorsal motor nucleus of the vagal nerve (DMV) and extending to the midbrain and other brainstem regions in later stages of the disease. The vagal nerve receives projections from the enteric nervous system (ENS) and the spinal cord and is proposed as the connection between the peripheral nervous system (PNS) and the CNS [90].

The majority of PD patients also display nonmotor symptoms such as dysphagia, constipation, and gastroesophageal reflux [91–93]. These symptoms that may reflect a prodromal stage of the disease appear to coincide with gastrointestinal pathology involving the deposition of phosphorylated *α*-synuclein in enteric neurons [94]. Histological analyses have shown that the PNS and the gastrointestinal (GI) tract are affected by both pathological and inflammatory events in early stages in PD [90, 93, 95]. Intestinal hyperpermeability along with increased exposure to intestinal bacteria and bacterial endotoxins, oxidative stress (nitrotyrosine), and *α*-synuclein deposits have been observed in the intestinal mucosa of patients suffering from PD using immunohistochemical and serological analyses [95]. Moreover, recent studies have reported alterations in the composition of the gut microbiota in patients with PD [96]. Although the precise nature of these alterations has not been identified to date, these findings suggest that gut microbiota might play a role in PD pathology.

It has been suggested that prion-like cell-to-cell transmission through the vagal nerve and spinal cord may require the involvement of the immune system [97]. The presence of exogenous pathogens in the GI tract may lead to the activation of macrophages, which consequently secrete inflammatory mediators inducing oxidative stress and affecting the surrounding tissues and consequently initiating the synucleinopathy in the ENS. These events may influence the intestinal function as evidenced by the premotor symptoms. Cell-to-cell transmission of misfolded *α*-synuclein aggregates through the ENS into the brain may further contribute to the sustained activation of local macrophages as well as the progression of the pathology. Further research is necessary to unravel the involvement of the ENS and the role of the immune system in disease initiation and propagation towards the CNS.

## 7. Immunomodulatory Strategies for PD

Considering the potential role of neuroinflammation in the initiation and progression of PD, targeting the immune system is a promising strategy for treating PD. Several studies targeting the inflammatory pathways mediated by microglial cells have been carried out. Nigral overexpression of dominant negative TNF in the 6-OHDA rat model attenuated microglial activation and resulted in decreased neuronal degeneration and improved motor behavior [60, 98]. Therapeutic approaches with the aim of modulating the peripheral immune system have been designed to trigger T cells *in vivo* upon exposure to distinct compounds and transfer them to preclinical models of PD. For example, Benner and coworkers reported that adoptive transfer of T cells immunized with glatiramer acetate (a synthetic random amino acid polymer used as an immunization-based antigen) to MPTP-treated mice led to the infiltration of T cells in the SN, suppressed microglial activation, and increased synthesis of astrocyte-associated glial cell line-derived neurotrophic factor, resulting in neuroprotection of dopaminergic neurons [99]. NSAIDs, such as aspirin, salicylic acid, and ibuprofen, have been shown in certain studies to have neuroprotective effects on dopaminergic neurons and have been suggested as a preventive treatment for PD [10, 48, 49, 100]. However, more research is necessary on the possible correlation between the use of anti-inflammatory drugs and developing PD. Additional anti-inflammatory compounds, like naloxone, minocycline, pioglitazone, and FK506 have been shown to reduce microglial activation and neuronal cell death in different models of PD [13, 58, 101, 102].

The recently described transmissible nature of *α*-synuclein underlying the progression of the disease has opened new possibilities for therapeutic strategies [89]. In an attempt to target (extracellular) *α*-synuclein, two immunotherapeutic strategies have been explored: active immunization using the patient's own immune system to generate antibodies against *α*-synuclein or passive immunization using the direct administration of antibodies against different domains of *α*-synuclein. Masliah et al. pioneered experimental active immunization targeting *α*-synuclein. They reported that immunization of WT *α*-synuclein transgenic mice with *α*-synuclein decreased protein accumulation in neuronal cell bodies and synapses and reduced neurodegeneration [103]. Later, Sanchez-Guajardo et al. used a viral vector-based *α*-synuclein rat model to test the active immunization against *α*-synuclein. Vaccination with recombinant *α*-synuclein 6 to 10 weeks before intracerebral injection of *α*-synuclein reduced protein inclusions in the SN associated with an increase in CD4^+^ T cells and microglial activation [104]. Another vaccination-based approach consisted of administrating short fragments of *α*-synuclein conjugated to a carrier, also known as the AFFITOPE® AFF1 vaccine, which resulted in the stimulation of antibodies against the C-terminal part of *α*-synuclein in two mouse models of PD [105]. This strategy has been reported to reduce neuropathology and increase microglial activation and anti-inflammatory cytokine expression. Based on these preclinical results, AFFITOPE vaccines are in a phase I clinical trial for PD and a phase II trial for multiple system atrophy (MSA). Masliah and coworkers also conducted the first study implementing passive immunization. Passive immunization gives the possibility to reduce the dose or to stop the treatment in case of adverse effects. They showed that passive immunization with a novel monoclonal antibody against the C-terminus of *α*-synuclein (9E4), able to cross the BBB, improved *α*-synuclein pathology and motor symptoms [106]. These observations support the idea that passive immunization with monoclonal antibodies might be therapeutically relevant for PD and other neurodegenerative disorders. It has been postulated that the antibody-mediated clearance of extracellular *α*-synuclein mainly occurs in microglia through the Fc*γ* receptor as shown by the increased localization of *α*-synuclein as well as the antibody in microglia [107]. A phase Ib trial is currently ongoing to evaluate the humanized form of 9E4, called PRX002, and assess the safety and pharmacokinetics in patients with idiopathic PD. Fagerqvist et al. generated antibodies against different *α*-synuclein conformations (oligomeric or protofibrillar *α*-synuclein). This resulted in a decrease of these possible toxic species in the mouse brain as well as in human postmortem brain samples [108]. Although preclinical studies assessing both immunization strategies have been successful, further research is warranted to design and investigate *α*-synuclein conformation-specific antibodies.

## 8. Conclusions

Neurodegenerative disorders threaten our society with a substantial economic burden. There is an urgent need to develop novel therapeutic strategies acting on the underlying disease pathogenesis in order to slow down or halt disease progression. In this regard, it is of uttermost importance to better understand how neuroinflammation plays a role in the initiation and progression of PD. Animal models and human studies have generated multiple evidence for the involvement of microglia and T lymphocytes in PD; however, their specific role in disease initiation and progression remains elusive. Immune alterations in response to different *α*-synuclein conformations may play a critical role in modulating disease progression and outcome. Identifying the immuno-pathogenic conformational state of *α*-synuclein might open novel therapeutic options. The recruitment of peripheral monocytes has been reported to contribute to PD-associated neurodegeneration, but the exact role of these peripheral monocytes in the disease process remains to be determined. Further insights into the *α*-synuclein pathology occurring in the CNS or in the ENS as well as the role of the immune cells in this process will be particularly important considering early therapeutic interventions. Recent findings concerning the involvement of LRRK2 in microglial and monocytic activation may provide valuable information about its interactions with *α*-synuclein and the link to neuroinflammation in PD. In conclusion, targeted interventions aiming at modifying the pathological immune response in PD may prove efficient in slowing disease progression. Future research should focus on identifying new drug targets by broadening our understanding of neuroinflammatory processes in PD-associated disease initiation and progression.
